# Bacterial therapy: a promising strategy for cancer immunotherapy

**DOI:** 10.20892/j.issn.2095-3941.2023.0292

**Published:** 2023-11-14

**Authors:** Yinsong Wang

**Affiliations:** The Province and Ministry Co-sponsored Collaborative Innovation Center for Medical Epigenetics, Key Laboratory of Immune Microenvironment and Disease (Ministry of Education), Tianjin Key Laboratory on Technologies Enabling Development of Clinical Therapeutics and Diagnostics (Theranostics), School of Pharmacy, Tianjin Medical University, Tianjin 300070, China

Cancer immunotherapy is capable of stimulating the body’s immune system to selectively attack cancer cells and has the advantages of high efficacy and low toxicity over traditional therapies. Numerous immunotherapies have been approved for clinical applications or clinical trials, including immune checkpoint blockade, adoptive cell therapy, recombinant cytokines, bispecific T-cell engagers, and cancer vaccines^1^. Among these therapies, checkpoint blockade of programmed death protein 1 (PD-1) and its ligand, programmed death-ligand 1 (PD-L1), has successfully shifted the clinical landscape, but few patients benefit due to low tumor immunogenicity^2^. Furthermore, immunotherapy targeting a single mechanism often leads to acquired resistance in patients, and thus weaken the therapeutic efficacy. An increasing body of evidence has demonstrated that combining multiple mechanisms of immune stimulation or targeting multiple pathways of immune escape are more efficient in maintaining the tumor-immune cycle and restoring the body’s anti-cancer immunity^3,4^.

Bacterial therapy, which utilizes inactivated, attenuated, or killed bacteria to provoke the immune system *via* multiple mechanisms and pathways has long been applied to fight cancers. Dating back to the 19th century, William Coley injected heat-inactivated *Streptococcus* and *Serratia marcescens* (the so-called Coley toxin) intratumorally and unexpectedly observed tumor regression. For this reason, William Coley is hailed as the father of cancer immunotherapy^5^. Many cancer patients refractory to standard therapy benefited from this novel therapeutic strategy, resulting in partial or complete cancer regression. Another successful example of bacterial therapy involves the FDA-approved Bacillus Calmette-Guerin vaccine, which is derived from an attenuated variant of *Mycobacterium bovis*, for bladder cancer treatment in 1990^6^. Due to a lack of understanding the mechanisms underlying anti-cancer action, bacterial therapy has not been widely applied in clinical practice. With the gradual recognition of a relationship between cancer and the host immune system, as well as the rapid development of synthetic biology and material science, bacterial therapy has gained renewed interest in the field of cancer research^7^.

## Advantages of bacterial therapy

**Figure 1:**
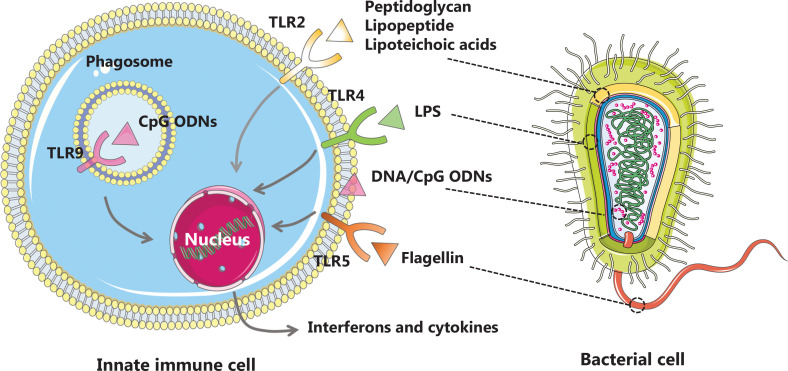
Schematic illustrating the activation of innate immune cells through recognition of bacterial PAMPs *via* PRRs (TLRs).

One of the leading advantages of bacterial therapy is that bacterial therapy can efficiently stimulate anti-tumor immune responses *via* multiple mechanisms. The immune modulation mechanisms underlying bacterial therapy are illustrated in **Figure 1**. Owing to the presence of pathogen-associated molecular patterns (PAMPs), mainly including lipopolysaccharides (LPSs), peptidoglycans, lipoteichoic acid, flagellin, and nucleic acids (DNA and RNA), bacterial therapy can activate innate immunity to fight cancer or facilitate cancer treatment by binding to pattern recognition receptors (PRRs), such as Toll-like receptors (TLRs), expressed by dendritic cells (DCs), macrophages, monocytes, and B lymphocytes^8^. LPS, the major component of the outer membrane of Gram-negative bacteria, promotes the maturation of DCs and M1 polarization of macrophages by activating TLR4-mediated signaling pathways^9^. Flagellin can be specifically recognized by TLR5 on antigen-presenting cells, and thus activate immune and inflammatory responses to influence tumor progression^10^. As specific TLR2 agonists, peptidoglycans, lipopeptides, and lipoteichoic acid from Gram-positive bacteria modulate the phenotypes and functions of DCs, macrophages, natural killer cells, and T cells to initiate anti-cancer immune responses^11^. Cytosine-guanine oligodeoxynucleotide (CpG ODN) motifs in bacterial and synthetic DNA are potent TLR9 agonists and have been used as vaccine adjuvants in clinical trials to promote antigen presentation and elicit anti-cancer T cell responses^12^.

In addition to adjuvant functions, bacteria have predominant hypoxia targeting and tumor colonization abilities, also making adjuvants an ideal candidate for cancer treatment. Hypoxia is a common feature of solid tumors and is recognized as one of the major reasons for poor clinical outcomes. Many investigations have shown that tumor hypoxia is closely associated with chemotherapy and radiotherapy resistance. Thus far, targeting hypoxia remains a great challenge for cancer therapy^13^; however, the hypoxic regions of tumors provide an appropriate environment for anaerobic bacteria to colonize and proliferate. Several bacterial strains, such as *Salmonella*, *Escherichia coli*, *Clostridium*, and *Listeria*, have been shown to possess inherent oncolytic activity by invading and colonizing solid tumors. Bacterial strains also serve as bioactive carriers to selectively deliver therapeutic agents or in combination with other therapies to acquire synergistic anti-cancer efficacy^14^. Another important advantage of bacterial therapy is the genetic modifiability of bacteria, meaning that bacteria can be easily engineered using the tools of synthetic biology. Bacteria are genetically engineered for attenuation or production of specific protein toxins, tumoricidal agents, and tumor antigens, which enhances therapeutic efficacy and reduces toxicity^15^. However, the biosafety of genetically engineered bacteria is the key issue currently facing clinical applications for cancer immunotherapy.

## Engineered nanomaterials for bacterial therapy

Apart from genetic engineering, utilizing the advantages of nanomaterials to improve bacterial immunotherapeutics has also received increasing attention in recent years. Surface modification of nanomaterials has been applied to enhance the biosafety of live bacteria and introduce other therapeutic functions but without influencing bacterial intrinsic properties^14,15^. In addition, biomimetic nanotherapeutics based on bacteria-derived components integrate the biological properties and functions of bacteria with the controllability and versatility of nanomaterials, thereby bringing a new vitality in the area of cancer treatments. More importantly, bacteria-derived components may be safer than live bacteria for clinical applications. Outer membrane vesicles (OMVs) are released spontaneously from Gram-negative bacteria and have spherical vesicle-like nanostructures, 10–300 nm in size. OMVs have been widely utilized to develop biomimetic nanotherapeutics^16^. OMVs are rich in bioactive components (LPS, phospholipids, membrane proteins, nucleic acids, and other products) and exhibit important biological functions, including interactions among bacteria, the host, and environment, and modulating immune responses^17^. In addition to native characteristics, OMVs have emerged as a promising nanomaterial for cancer combination treatments due to a strong loading capacity for a great variety of therapeutic agents. In a recent study reported by Li et al.^18^, OMVs were used as an mRNA delivery platform for personalized mRNA tumor vaccination by surface engineering of an RNA-binding protein (L7Ae). The results revealed that OMV-L7Ae rapidly adsorbs boxC/D sequence-labeled mRNA antigens *via* L7Ae-boxC/D binding and delivering the antigens into HEK-293T and DCs. We first developed a cellular nanofabrication technique involving prepared nanoscaled red blood cells for breast cancer treatment^19^. In our recent investigation^20^ we exploited this novel technique to fabricate bacterial nanomedicine that combines photodynamic-immunotherapy and chemotherapy for oral squamous cell carcinoma treatment.

## Opportunities, challenges and perspectives of bacterial therapy

Bacterial therapy has significant advantages over traditional cancer therapies and brings hope to refractory cancer patients. For example, the first bacterial oncolytic drug, *Clostridium ghonii* spore freeze-dried powder for injection, was developed by Shandong Xinchuang Biotechnology Co. Ltd. and has entered a phase II clinical trial in China for patients with advanced soft tissue sarcoma. Currently, the major challenge facing the clinical applications of bacterial therapy is reducing toxicity and promoting the specificity and therapeutic efficacy. Bacterial therapy based on genetically engineered bacteria is limited by low expression of target proteins, uncontrollable mutations, and unstable long-term effects. With the rapid development of synthetic biology strategies, e.g., modulating gene copy number, promoter strength and bacterial metabolic rate, it is possible to resolve these potential problems. Anaerobic bacteria are prone to colonize in the hypoxic necrosis area of tumor tissues and subsequently cause spatial heterogeneity of infiltrating immune cells, resulting in less satisfied anti-cancer effects. Combining bacterial therapy with other therapies that consume oxygen, such as photodynamic therapy, sonodynamic therapy, and radiotherapy, is expected to achieve synergistic anticancer effects. With respect to the application issues, such as the time-consuming task of extracting bacterial components and poor patient compliance stemming from multiple injections, orally-administered bacteria serving as a “living factory” to produce “products” may be an optimal strategy. In summary, it is possible to develop bacterial therapy meeting the above requirements using synthetic biology tools and nanomaterials. Elaborately engineered bacteria will work together with immune cells to fight cancers more efficiently, adding a new dimension to cancer immunotherapy.
